# Complications and surgical treatment after pathological fracture associated to HIV secondary disease. A case report

**DOI:** 10.1093/jscr/rjac223

**Published:** 2022-06-10

**Authors:** Jorge Fuentes-Sánchez, Enrique Gómez-Barrena

**Affiliations:** Orthopaedic Surgery and Traumatology Department, Hospital Universitario La Paz, P° Castellana 261, 28046 Madrid, Spain; Orthopaedic Surgery and Traumatology Department, Hospital Universitario La Paz, Universidad Autónoma de Madrid, P° Castellana 261, 28046 Madrid, Spain

## Abstract

Due to advances in retroviral treatment, human immunodeficiency virus (HIV)-related disease may become chronic and the patient survival has substantially increased. Osteoarticular disease in those patients include multifocal osteonecrosis and its complications. Pain and functional limitation may be due to these complications, frequently underdiagnosed, including pathological fractures. Its prompt management may require a different approach than osteosynthesis. We present a long-term chronic HIV patient with severe pain and limitation. A tibial plateau pathological fracture associated to multifocal osteonecrosis was identified and treated with osteonecrosis debridement and total knee arthroplasty (TKA). Acute periprosthetic joint infection developed and required debridement, antibiotic and implant retention. The contralateral knee, also with multiple osteonecrosis foci, was managed with early TKA. We highlight the importance of timely surgical reconstruction to avoid serious limitation and complications.

## INTRODUCTION

Infection due to human immunodeficiency virus (HIV) has evolved from a rapidly progressive fatal disease into a chronic disease, increasing the long-term osteoarticular complications. Most commonly, it includes osteopenia, osteonecrosis, degenerative joint disease and even pathological fractures, [1, 2] in patients at increased risk of osteoarticular infection [3]. Joint involvement may produce significant functional disability in these patients [4]. Fractures, frequently underdiagnosed if slowly developing, are usually managed through osteosynthesis [10–14]. However, late diagnosis may produce a more complex deformity and functional disability, and the management approach may vary.

This case is illustrative of how arthroplasty can be a useful tool in HIV patients, even during the acute onset of pain and disability secondary to a pathological fracture without associated trauma. In addition, complications such as infection need careful risk assessment. Finally, the diagnosis of severe multifocal osteonecrosis in the contralateral knee raises the issue of adequate surgical timing to prevent a catastrophic collapse.

## CASE REPORT

A 47-year-old female patient presented with a history of HIV infection diagnosed 20 years ago, CDC-C2 stage due to multiple recurrent pneumonia with hospital admissions, former parenteral drug addict, chronic liver disease due to hepatitis C virus (HCV), type 2 diabetes and polyneuropathy. Also, the patient sustained a treated oncologic history of both *in situ* cervix and glottis cancer. She received treatment for her HIV infection through multiple regimes of highly active antiretroviral therapy (HAART) with undetectable viral load for 10 years.

**Figure 1:**
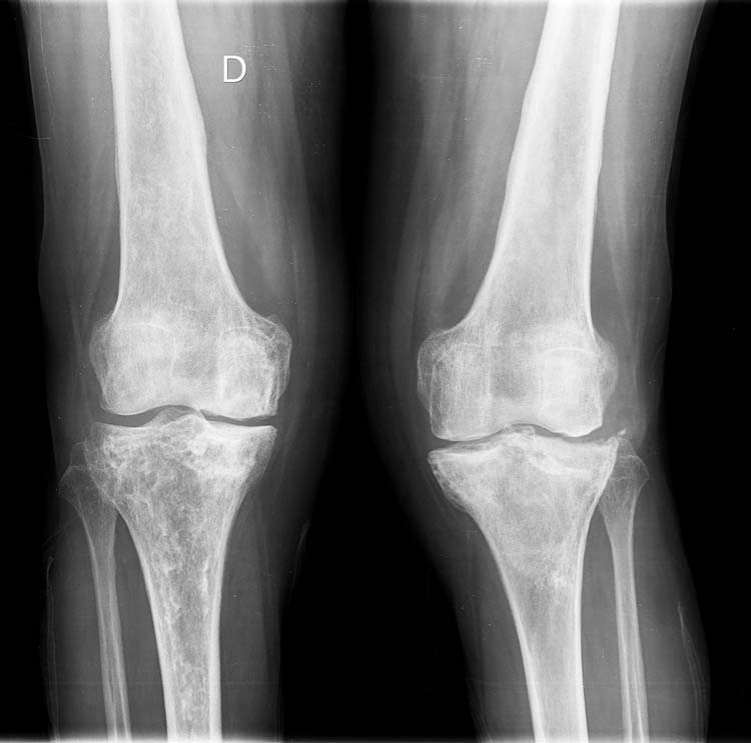
Initial both-legs standing radiographs of the knee. It showed a pattern of diffuse osteopenia with areas of sclerosis, lateral extended bone reaction and decreased lateral articular space (Kellgren–Lawrence 3) in the left knee. Bone necrosis with decreased medial articular space (Kellgren–Lawrence 3) were observed in the right knee.

She was referred to our Service to evaluate progressive and disabling pain in both knees. Physical examination revealed bilateral quadriceps atrophy with valgus laxity in the left knee and varus laxity in her right knee. Range of motion (ROM) in both knees was 0°–100°. Standing radiographs showed a pattern of diffuse osteopenia and necrosis (Fig. 1).

**Figure 2:**
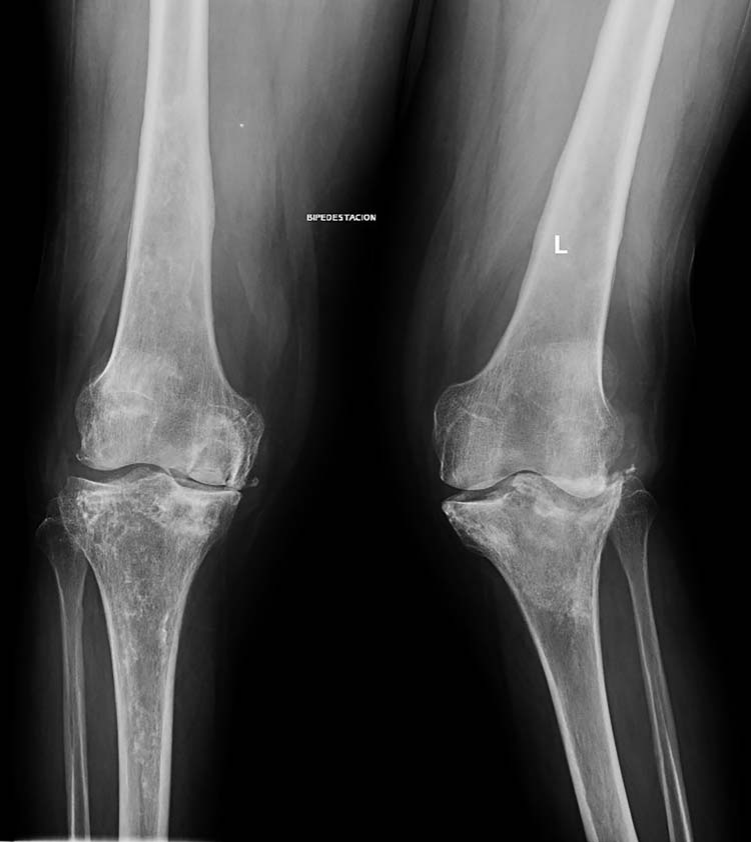
Standing X-rays of the left knee 8 months later showed collapse with depression of the external tibial plateau and associated bone defect that caused 28° valgus deformity.

At this point, the patient was recovering from a respiratory infection with multiple admissions and relapses, with CD4 cell count of 146 cells/ml. Therefore, conservative treatment was recommended, but pain control was insufficient. After 8 months she was admitted to the emergency service in a wheelchair due to intractable acute pain without prior trauma in the left knee, which prevented weight-bearing. New standing X-rays of the left knee showed collapse of the external tibial plateau (Fig. 2). At this time, the patient exhibited acceptable medical control, with CD4 240 cells/ml and undetectable viral load.

**Figure 3:**
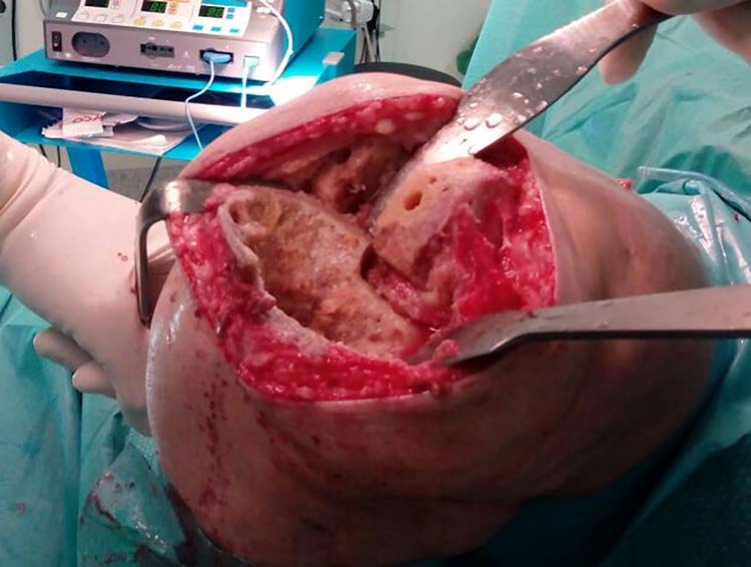
Surgical photograph showing the large bone defect.

**Figure 4:**
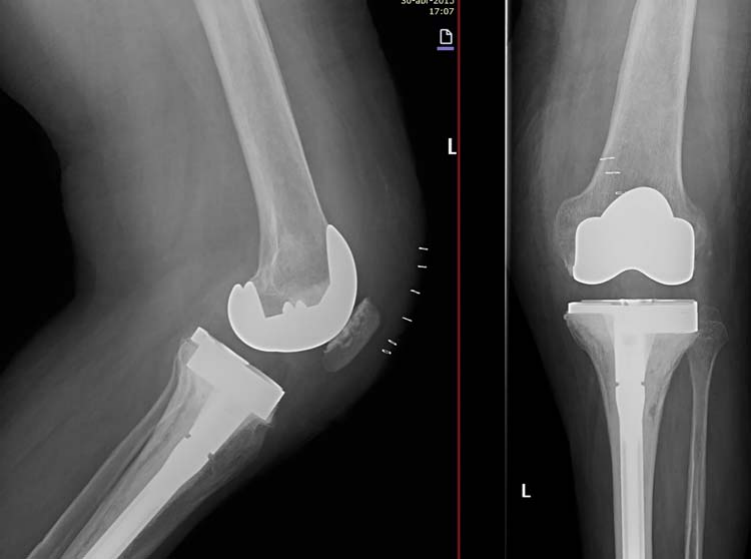
X-ray of the left knee showing reconstruction with 10-mm tibial lateral block and a tibial diaphyseal stem.

**Figure 5:**
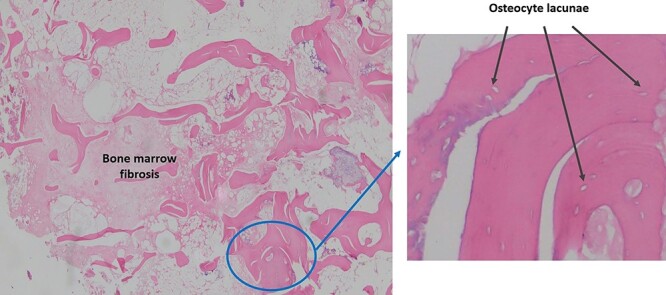
Pathological sample 1: bone marrow fibrosis on the left and osteolysis with empty lacunae enlarged in perimeter without osteocytes on the right.

**Figure 6:**
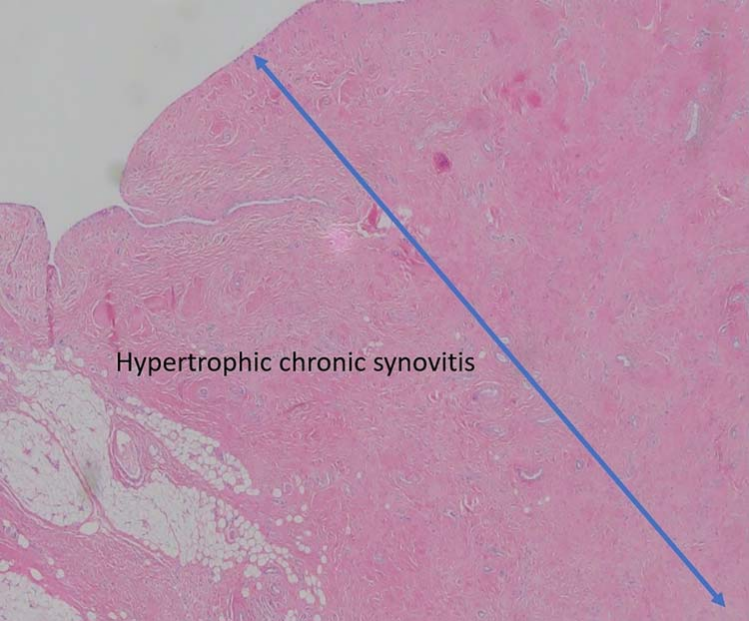
Pathological sample 2: hypertrophic chronic synovitis, without signs of acute inflammation, evidencing a long evolution of the process.

Due to the poor bone quality and defect associated to osteonecrosis (Fig. 3), osteosynthesis was discarded and total knee arthroplasty (TKA) was performed with lateral tibial block and diaphyseal stem (Fig. 4). Microbiological samples confirmed the absence of infection. Pathological samples confirmed osteolysis and chronic synovitis with osteoporosis and osteonecrosis, leading to the pathological fracture (Figs 5 and 6).

At 3 weeks postoperatively, our patient suffered from an acute periprosthetic joint infection. Methicillin-sensitive *Staphylococcus aureus* (MSSA) was isolated and successfully treated through debridement, antibiotic and implant retention.

**Figure 7:**
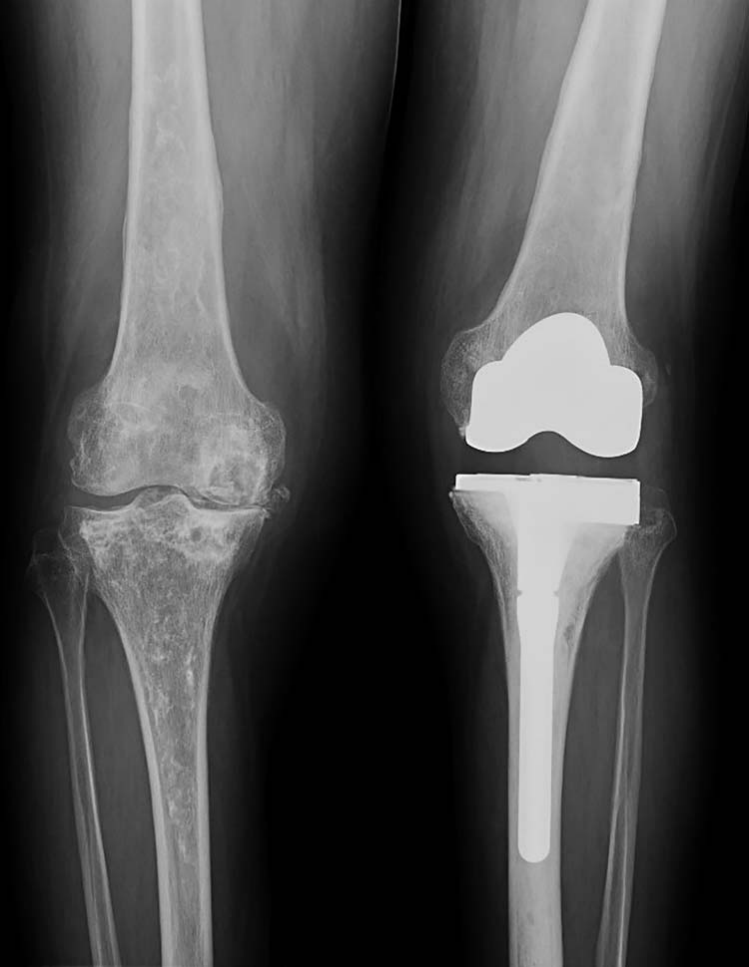
Evolution of the right knee by standing radiographs during follow-up with progression toward joint space disappearance (Kellgren–Lawrence 4) in a severe varus deformity.

**Figure 8:**
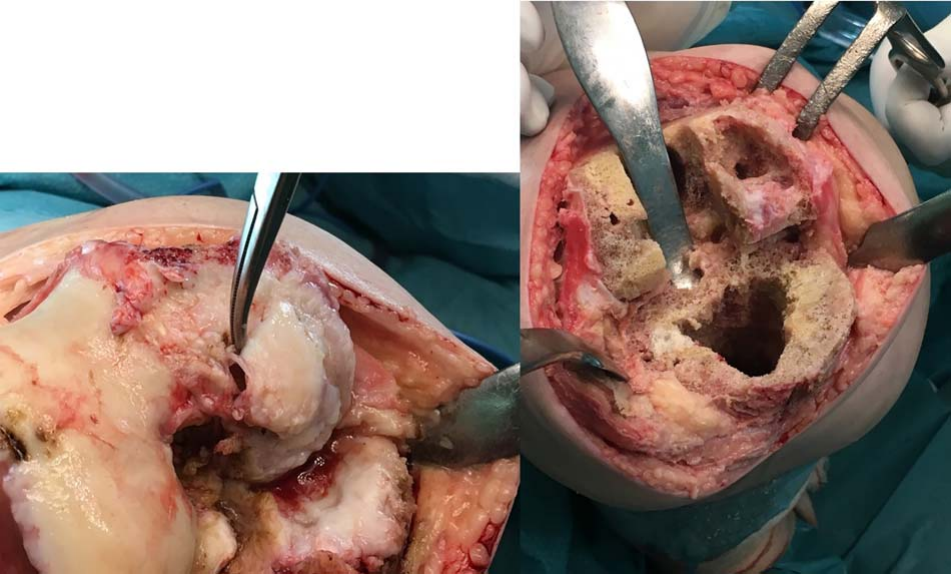
Surgical photograph showing the large bone defect in the right knee especially in the femoral condyles.

In the subsequent follow-up, pain progressively increased in the contralateral knee. Standing X-rays confirmed a severe deterioration of the right knee (Fig. 7). Given the bone defect and the consequent instability (Fig. 8), a CCK-type prosthesis was required, including medial femoral condyle blocks. The microbiological results were again negative, whereas the pathology results confirmed both severe osteoporosis and osteonecrosis.

**Figure 9:**
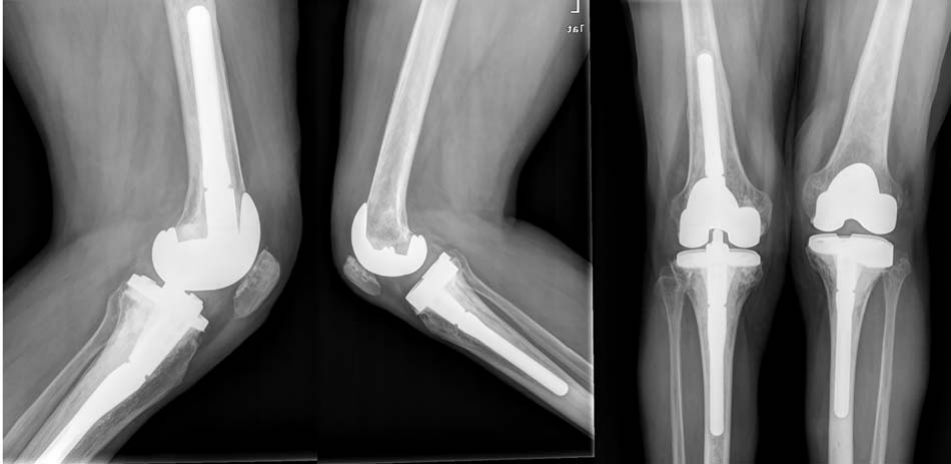
Both knees standing radiographs at 4-year follow-up with no signs of radiological loosening.

In the early postoperative period, no complications were detected, with pain decrease. After 4 years, the patient’s active ROM was 0–110° bilaterally, with no signs of radiological loosening (Fig. 9) and no pain, leading an unrestricted walking daily life without aids.

## DISCUSSION

In HIV patients, comorbidities and HAART play a fundamental role in the evolution of joint pathology [2]. Besides, the associated fracture risk increases in patients with HIV and HCV coinfection in parallel to the risk of secondary infection, as seen in our patient [8].

In such a complex case, presenting with an acute pathological tibial plateau depression fracture (Schatzker III), the first option would have been elevation and osteosynthesis. However, arthroplasty was the selected treatment given the poor bone quality. In the literature, there is no clear information about how to treat this type of fracture in such a patient. However, the risks inherent to TKA have been thoroughly studied, [4–6, 9] and there is an intrinsic risk of septic arthritis, with MSSA as the main etiological agent, as occurred in our patient [3]. Due to the whole set of non-negligible comorbidities, ensuring the patient has definitively abandoned any intravenous drug is a pre-requisite to optimize the intervention.

For the first surgery, and due to her functional limitation and uncontrolled pain, preoperative optimization could not be fully carried out. However, at the time of the early second surgery to avoid a similar contralateral collapse, favorable preoperative analytical and clinical parameters were reached. Lifestyle advice and pharmacological treatment provide the first-line treatment to improve bone health [7].

These measures are justified since the risks include not only the fracture but also other potential underlying complications, and particularly infection. Although a consensus is well established, a recent meta-analysis regarding infection secondary to fracture treatment in HIV patients did not obtained significant results [10]. This excludes open fractures, where Aird *et al*. [11] found an increased risk of infection with CD4 values < 350.

Some observational studies and a systematic review have corroborated that surgical management in HIV closed fractures should not be avoided, regardless of the CD4 [12, 13, 15]. A recent study proposed that CD4 values may not be a limiting factor but rather the set of accompanying factors. Therefore, one must counterbalance an increase in complications depending on the clinical category of HIV, particularly in the case of open fractures, and the number of CD4 [14].

Our patient laboratory analysis, being at CDC-C2 stage albeit with a closed fracture, showed 240 cells/ml as the presurgical CD4 count at the first TKA (suffering from an early PIJ), whereas the values before the second surgery (with no subsequent infection) were 320 cells/ml.

When considering the surgical management, joint reconstruction needs planning. Stemmed, cemented, constrained arthroplasty techniques must be ready for use in these patients. Presurgical CD4 counts, together with bone quality due to osteonecrosis and osteoporosis, must be taken into account in these patients. Adequate surgical timing, both medically and surgically, is paramount to decrease complications and maximize the short- and long-term outcome.
